# The Effects of a Reading-Based Intervention on Emotion Processing in Children Who Have Suffered Early Adversity and War Related Trauma

**DOI:** 10.3389/fpsyg.2021.613754

**Published:** 2021-03-24

**Authors:** Julia E. Michalek, Matteo Lisi, Deema Awad, Kristin Hadfield, Isabelle Mareschal, Rana Dajani

**Affiliations:** ^1^Department of Biological and Experimental Psychology, Queen Mary University of London, London, United Kingdom; ^2^Department of Psychology, University of Essex, Colchester, United Kingdom; ^3^School of Psychology, Trinity College Dublin, Dublin, Ireland; ^4^Biology and Biotechnology Department, Hashemite University, Zarqa, Jordan; ^5^Jepson School of Leadership Studies, University of Richmond, Richmond, VA, United States

**Keywords:** war trauma, refugee children, emotion recognition, reading intervention, affective development

## Abstract

Early adversity and trauma can have profound effects on children’s affective development and mental health outcomes. Interventions that improve mental health and socioemotional development are essential to mitigate these effects. We conducted a pilot study examining whether a reading-based program (*We Love Reading*) improves emotion recognition and mental health through socialization in Syrian refugee (*n* = 49) and Jordanian non-refugee children (*n* = 45) aged 7–12 years old (*M* = 8.9, 57% girls) living in Jordan. To measure emotion recognition, children classified the expression in faces morphed between two emotions (happy–sad and fear–anger), while mental health was assessed using survey measures of optimism, depression, anxiety, distress, and insecurity. Prior to the intervention, both groups of children were significantly biased to interpret ambiguous facial expressions as sad, while there was no clear bias on the fear–anger spectrum. Following the intervention, we found changes in Syrian refugee children’s bias in emotion recognition away from sad facial expressions, although this returned to pre-intervention levels 2 months after the end of the program. This shift in the bias away from sad facial expressions was not associated with changes in self-reported mental health symptoms. These results suggest a potential positive role of the reading intervention on affective development, but further research is required to determine the longer-term impacts of the program.

## Introduction

More than 1% of the world’s population is currently forcibly displaced: 26 million of the 79.5 million forcibly displaced people are refugees, and more than half of these are under the age of 18 (UNHCR, 2020). Reports from conflict zones show a staggering exposure to traumatic war events amongst refugee children (Goldstein et al., 1997; Ahmad et al., 2000; Allwood et al., 2002; Morgos et al., 2008; Panter-Brick et al., 2009; Sirin and Rogers-Sirin, 2015) that can lead to complex and challenging physical and mental health issues (Lindert et al., 2009; Reed et al., 2012; Kane et al., 2014; Dyregrov et al., 2015; Slobodin and De Jong, 2015; Wylie et al., 2018). Further, displacement forces children to leave their homes and, in many cases, to separate from their family. Displaced children also often experience a lack of basic necessities, such as food, medicine, and provisions (Kuterovac et al., 1994; Allwood et al., 2002; Panter-Brick et al., 2009).

Refugee children, exposed to trauma, displacement, and poverty, are at increased risk for various emotional and mental health problems, such as depression and PTSD (Henley and Robinson, 2011), and possibly vulnerable to poorer executive functioning (Chen et al., 2019; Mirabolfathi et al., 2020). These children often display increased levels of psychological distress and behavioral problems, with a higher risk of developing chronic psychopathology than non-refugee children (Derluyn et al., 2004; Lindert et al., 2009). For example, children who have been forcibly displaced as a result of war display increased rates of emotional and behavioral disorders (the most frequent being PTSD, anxiety, sleep disturbances, depression and grief) and many of these children experience high psychopathological comorbidity (Henley and Robinson, 2011). According to screening questionnaires and clinical evaluations, rates for these disorders amongst refugee populations can be as high as 50% for PTSD and 40% for depressive symptoms (Thabet et al., 2002; Alpak et al., 2015; Sirin and Rogers-Sirin, 2015; Ozer et al., 2016). Even after resettlement, refugees often face substantial challenges, such as racial discrimination and harassment, poverty, and language barriers (Sujoldzic et al., 2007; Fazel et al., 2012; Fazel, 2018). Recent evidence by A. Chen et al. (2019) suggests that poverty is associated with working memory deficits in both refugee and non-refugee children over and above any effects of trauma. This study highlights the importance of current living conditions on healthy development in both children who have and have not been exposed to war and displacement.

Childhood and early adolescence are critical periods for the development of interpersonal skills and good decision-making strategies (Steinberg and Morris, 2001). Emotion recognition, the foundation of communication and social interactions, conveys critical information about state of mind. The inability to interpret emotional expressions is associated with mental health disorders of anxiety, depression, and post-traumatic stress disorder (PTSD) (Bourke et al., 2010; Demenescu et al., 2010; Milanak et al., 2018; Rutter et al., 2019). For example, an attentional bias for angry compared to neutral facial expressions has been shown in children with behavioral inhibition (Pérez-Edgar et al., 2011), a temperament trait characterized in early childhood by hypervigilance and avoidance to novel stimuli (Marshall et al., 2009), and previously linked to internalizing, avoidant behaviors, and anxiety disorders (Fox et al., 2005; Chronis-Tuscano et al., 2009). Reeb-Sutherland et al. (2015) found that behaviorally inhibited adolescents who are suffering from social anxiety disorder exhibited over-identification of fearful faces compared to angry faces, suggesting an important role of emotion processing in anxiety disorders.

However, in other reports, the association between impaired emotion recognition and psychopathology is less clear. For instance, depressed adolescents did not show deficits in emotion processing accuracy in a recent study (Schepman et al., 2012). Similarly, social anxiety disorder was not linked to differences in emotion recognition tasks (Pepper et al., 2018). Furthermore, although impairments in emotion recognition from facial expressions were found in adults with anxiety and adults with depression, these abnormalities were not found in children with anxiety disorder (Demenescu et al., 2010).

Childhood adversity is also a strong predictor of many mental health problems, including the occurrence of depression and anxiety symptoms (Kendler et al., 2002; Espejo et al., 2007; Brown et al., 2008; Gonzalez et al., 2012; Nanni et al., 2012). There is evidence that childhood abuse alters brain structure as well as function, and behavior on cognitive and affective tasks, such as fear processing, autobiographical memory, emotion regulation, reward processing and risk taking, are atypical (McGowan et al., 2009; Kuehl et al., 2019). Childhood abuse has been linked to abnormalities in emotion processing and interpersonal skills (Herringa, 2017), and is associated with a tendency to over-identify negative emotions. Abused children are more accurate at recognizing angry faces (Pollak et al., 2000; Pollak and Kistler, 2002), they detect angry faces with less perceptual information (Pollak and Sinha, 2002), take longer to disengage from angry facial expressions (Pollak and Tolley-Schell, 2003), and show increased brain activation patterns in reaction to angry faces compared to age-matched controls (Pollak et al., 2001). Children exposed to early and ongoing psychosocial deprivation also show deficits in their emotion processing abilities, displaying a bias toward threat stimuli (Tottenham et al., 2010; Nelson et al., 2013; Troller-Renfree et al., 2015). It is also worth noting that emotion processing abnormalities, e.g., the inability to effectively regulate emotions, have been linked to both childhood maltreatment history and psychopathology (Berzenski, 2018), whereas healthy emotion regulation abilities are a predictor of resilience amongst children with a history of early life adversity (Curtis and Cicchetti, 2007).

Given these challenges, it is critical to study how refugee children’s mental health and emotion processing – vital for healthy social development – can be improved with potential cascading effects across the lifespan. Emotion socialization theory posits that children learn about emotions (interpreting them in others and regulating their own), through social contexts in schools and houses, by interactions with parents, teachers, and peers (Pollak and Thoits, 2008). It has been argued that family context specifically can impact emotion development and regulation in children in three different ways: through observation, parenting practices and behaviors, and the emotional climate of the family (Morris et al., 2007). This tripartite model emphasizes that children learn socialization and shape their emotion regulation abilities by observing emotional responses modeled by parents, from parental attitudes toward them and each other, as well as from parenting styles (Morris et al., 2007). It is important to note that parental influences are not proposed to simply shape children’s affective development, but rather to impact children’s emotion regulation abilities that mediate emotional development, suggesting a complex model of interactions between environmental factors and individual characteristics (Morris et al., 2007). These effects are not only prevalent in childhood but can also persist in adolescence and adulthood (Telzer et al., 2014; Cui et al., 2020; Speidel et al., 2020). Whilst emotion socialization in early childhood is predominantly influenced by family context, teachers and peers also play an important role in shaping emotion development in children (Eisenberg and Morris, 2002). Through emotion socialization, young children learn to identify and respond to affective states, understand their complexity, as well as to control and manipulate negative emotions (Cole et al., 2009). Therefore, social experiences at home and in school can shape emotion processing and regulation, and influence emotional intelligence (Pollak and Thoits, 2008; Garner et al., 1994). If access to these social experiences is impaired, emotion processing can be disturbed with the associated social and mental health problems described above. For instance, poor parental emotion socialization has been linked to abnormalities in both physiological and psychological reactions to stressors amongst adolescents and young adults (Guo et al., 2017). Similarly, negative parental emotional responses were linked to increased psychological distress in children, as retrospectively reported by young adults (Garside and Klimes-Dougan, 2002). Furthermore, families with clinically anxious preschool children were shown to exhibit poorer emotion socialization strategies compared to non-clinical control families (Suveg et al., 2005). A recent review has also indicated that classroom-based emotion socialization programs can have significant influence on children’s emotional competence (Valiente et al., 2020). Therefore, interventions that promote socialization could improve emotion processing with knock on improvements for mental health.

There are many interventions for refugee children, which have been shown to be successful at reducing PTSD symptoms (Brown et al., 2017; Morina et al., 2017; Fazel, 2018). The majority of these programs currently available for schools and communities focus on verbalization of past experiences (often employing forms of cognitive-behavioral or similar therapies) and various forms of creative arts therapies (Pacione et al., 2013; Tyrer and Fazel, 2014; Fazel and Betancourt, 2018). Such interventions have shown success in reducing anxiety and depression symptoms and increasing psychosocial and cognitive functioning (Jordans et al., 2010; Ruf et al., 2010; Ugurlu et al., 2016; Gormez et al., 2017). However, many of the available interventions were designed for adults or adolescents, and evidence-based interventions designed for younger refugee children are less common (Ehntholt and Yule, 2006; Jordans et al., 2009). Additionally, most interventions focus on symptom-reduction: much less is known about targeting psychological resilience and affective development – an approach which might be particularly beneficial to refugee children (Panter-Brick et al., 2018; Yaylaci, 2018; Foka et al., 2020). As the number of scalable, cost-effective interventions involving training lay volunteers, schoolteachers, and community workers increase and become more accessible (Fazel, 2018), it is important to develop programs which incorporate varied approaches and which are developed for younger children (Yaylaci, 2018; Frounfelker et al., 2020).

Interventions that affect socialization could improve mental health outcomes through children’s improved understanding of (their own and others’) emotional states. For example, group storytelling-based interventions could provide the context for socialization and some (e.g., Reach Out and Read [ROR]) show evidence for a positive influence of reading out loud on learning and prevention of developmental problems (Whitehurst et al., 1994; Zuckerman, 2009; Kalb and van Ours, 2014). The positive effects of shared reading not only improve cognitive development but may also promote children’s emotional wellbeing. For instance, language-based night-time routines (involving reading or singing with a parent) were associated with decreases in anxious, withdrawn, and aggressive behaviors in American pre-schoolers (Hale et al., 2011). However, these effects disappeared after controlling for other factors, such as parenting styles, and child and household characteristics, while another parental reading intervention for young Brazilian children (2–4 years old) showed no statistically significant improvements in socio-emotional competence, externalizing and internalizing behaviors, or aggression (Weisleder et al., 2018). These inconsistent findings could be due to differences in frequency, setting and duration of the programs, and could be influenced by children’s previous life experiences, as well as by the person delivering the intervention (e.g., parent vs. teacher or social worker). Some studies emphasize the positive effects of shared experiences through reading between parents and children, suggesting that attention and emotional engagement required in reading out loud could facilitate language skills acquisition as well as non-cognitive development (Sloat et al., 2015; Dowdall et al., 2019). Indeed, a recent report on literacy and wellbeing suggests that children and adolescents who show higher engagement with reading and writing have significantly better mental health outcomes as measured by life satisfaction, coping mechanisms, self-belief and improved mood (Killick and Bowkett, 2015; Clark and Teravainen-Goff, 2018).

Reading interventions targeting younger children (from infancy to primary school ages) also show positive outcomes on socio-emotional development. For instance, a reading intervention in South African children from low socioeconomic status (SES) backgrounds aged between 14 and 16 months, showed improvements in prosocial behavior compared to a control group, although these measures were taken only at follow up and thus the groups may have differed prior to the intervention (Murray et al., 2016). A classroom-based reading intervention in primary school children in Germany showed improvements in emotional vocabulary and emotional knowledge in participating children compared to controls (Kumschick et al., 2014). Similarly, a United States-based pilot study on a school-based program involving reading and socio-emotional learning showed positive effects on executive function as well as internalizing behaviors in kindergarten children at risk for emotional and behavioral disorders (Daunic et al., 2013). Reading-based programs for primary school-aged children can also lead to improvements in emotion processing and regulation. For example, a mediated-reading intervention in Chilean children (6–8 years old) showed larger short- and long-term improvements in their emotion recognition, empathy, and emotional regulation than a traditional-reading intervention (non-mediated), although children in the latter group also showed some improvements, which the authors attribute to an increase in their emotional competence (Riquelme and Montero, 2013; Riquelme-Mella and García-Celay, 2016). Indeed, non-mediated book reading programs could improve aspects of emotion socialization through role modeling. In line with the tripartite model of the emotion socialization theory, despite a lack of discussion about the emotional content of the stories, teachers and peers can influence children’s socialization through modeling emotional expressions during the reading sessions (observing others’ emotional displays and interactions). This type of social referencing and modeling, as well as positive emotional contagion (transmission of certain emotions or behaviors between individuals), could impact children’s affective development (Morris et al., 2007), and subsequently result in improved emotion recognition abilities. Suggestive of the idea that the impact of socio-emotional interventions might be influenced by children’s prior life experiences is Finlon et al.’s (2015) evaluation of an emotion-based preventative intervention aimed at increasing emotional intelligence through both classroom- and home-based exercises. They found that preschool children in the high-risk group – who had experienced more stress and less parental support – benefited more from the program compared to children in the low-risk group, indicating increased benefits for children with a history of early adversity.

In the present study, we examine whether a reading-based intervention (“We Love Reading”) could benefit children exposed to higher (i.e., refugees) and lower (i.e., non-refugees) levels of adversity, by increasing emotional knowledge and vocabulary as well as empathy (Kumschick et al., 2014; Riquelme-Mella and García-Celay, 2016). To do this, we used locally validated mental health questionnaires and computer-based emotion recognition tasks to measure children’s sensitivity to different facial expressions. We sought to answer the three following questions: (1) Is emotion processing affected in children who have been exposed to war and displacement? (2) Does a low-cost, reading-based intervention called We Love Reading (WLR) improve outcomes on emotion recognition or mental health measures in refugee and non-refugee children living in Amman? (3) How does children’s emotion recognition relate to survey measures of mental health?

We Love Reading is a reading program delivered by a Jordanian NGO (Taghyeer Foundation) in response to needs identified by the local community that trains local volunteers to deliver a story-telling program to children (ages 4–12) in weekly sessions. We Love Reading encourages women to read for a period of 2 months; however, due to school constraints and Ramadan, here the intervention was prepared by the NGO to be delivered over a shorter period of 5 weeks to test its effects. WLR is a community-led and scalable intervention that can be delivered in schools, houses, and community centers. Prior to participating in WLR, we expected that Syrian refugee children’s emotional competence would be altered, with refugee children showing a greater bias for perceiving sad and threat-related emotional expressions than Jordanian non-refugee children. We also expected that Syrian refugee children would have poorer mental health than their non-refugee peers, and that poorer mental health would be associated with atypicalities in emotion recognition. Following the WLR intervention, we hypothesized that children’s mental health, wellbeing, and emotion recognition would improve through emotion socialization, but that this would be more pronounced for the Syrian refugee children than the Jordanian non-refugee children.

## Materials and Methods

### Participants and Testing

**FIGURE 1:**
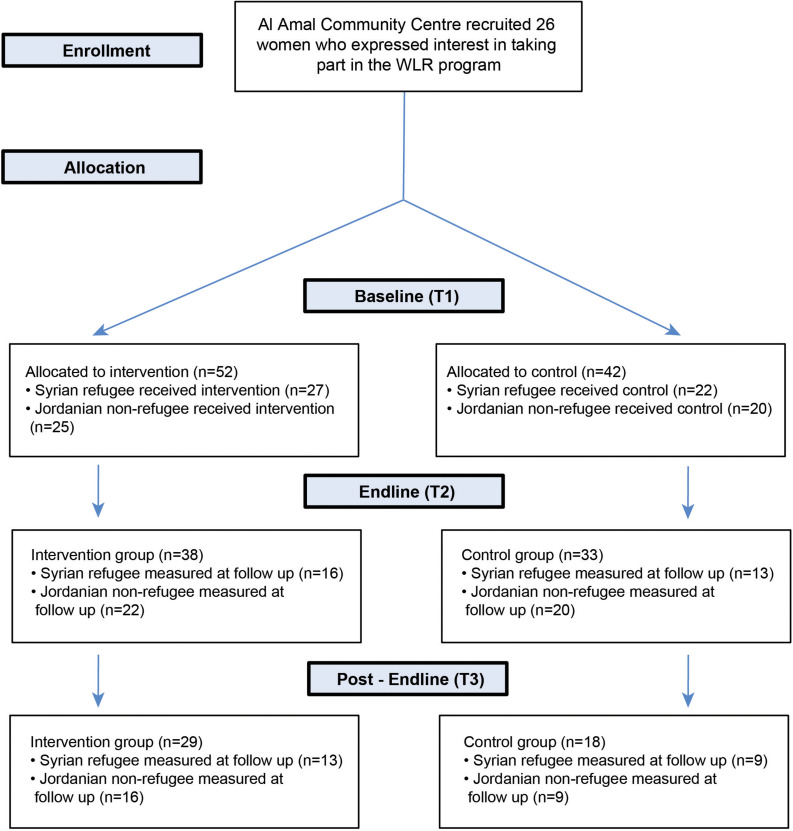
Participant flow chart.

Participants (*n* = 94) were Syrian refugee (*n* = 49, 30 girls) and Jordanian non-refugee (*n* = 45, 24 girls) schoolchildren aged between 7 and 12 years old (*M* = 8.9, *SD* = 1.3) living in Amman, Jordan, with testing over a 5-month period in 2019. 27 Syrian refugee and 25 Jordanian non-refugee children were allocated to the experimental group who took part in the WLR reading sessions, while 22 Syrian refugee children and 20 Jordanian non-refugee children were allocated to the control group (Figure 1). For the Jordanian non-refugee children, both reading sessions and testing took place in schools, with children from one school (Al Bonya, in the Sahab neighborhood) allocated to the experimental group while children from the other school (Al Shaqa ‘eq Al Nouman, in the Al Hashmi Al Shamali neighborhood) formed the control group. For the Syrian refugee children, reading sessions and testing took place in homes, with the neighborhood of Sweileh targeted for the intervention. A second neighborhood (Al Hashmi Al Shamali) was used to recruit children to the control group, where testing took place in a community center (Awael Al Khair). Control group participants did not participate in any of the reading sessions provided by WLR but instead continued to take part in their school and extracurricular activities as normal. All testing was conducted in Arabic and the only exclusion criterion for the children to participate in the research was age (children under 7 and over 12 years of age did not take part in the study).

The delivery and testing of the WLR program took place in different locations due to constraints of testing refugees in low- and middle-income countries, where inevitably some aspects cannot be controlled. Jordanian non-refugee children took part in WLR in schools, while Syrian refugees children took part in WLR at home or in community centers. This is because schools in Jordan are double shifted (Jordanian children in the morning and Syrian children in the afternoon), but most children and women preferred for WLR activities (program and testing) to occur in the mornings. Despite the different locations, the children were well-matched at the start of the intervention: they were all sampled from neighborhoods with high numbers of refugees, and the children did not vary on any measures of mental health (apart from, as expected, Trauma and PTSD) or on the emotion tasks at baseline.

We collected baseline data (questionnaires and emotion recognition tasks – see below) on 94 children at the start of the program in February 2019 (Timepoint 1), and re-collected questionnaire and emotion recognition task data on 71 children at the end of the program (2 months later, April 2019, Timepoint 2), and finally on 47 children 2 months after the end of the program in June 2019 (Timepoint 3) (Table 1). Whereas Syrian refugee children were tested in homes and a community center throughout the duration of the project, Jordanian non-refugees were tested in schools at T1 (baseline) and T2 (immediately after the intervention), and in homes at T3, due to summer holidays (see Table 2). We had substantial attrition between T2 and T3; this attrition similarly affected all four groups of children. Indeed, across our two follow up time points we found no evidence of a difference in the proportion of dropouts between Syrian refugee control and experimental groups [T2: χ^2^(1) = 7.3 × 10^–31^, *p* ≈ 1; T3: χ^2^(1) = 2.46 × 10^–31^, *p* ≈ 1], or between Jordanian non-refugee control and experimental groups [T2: χ^2^(1) = 1.00, *p* = 0.32; T3: χ^2^(1) = 0.95, *p* = 0.33]. Additionally, we found no differences at baseline in the emotion recognition bias, or in trauma and mental health measures between children who dropped out at T2 or T3 and children who remained, in both refugee and non-refugee groups (*p* > 0.05). Since the attrition was not related to group membership, the data at T2 and T3 can be considered as missing at random and their analysis should provide unbiased results. Furthermore, in order to maximize the sensitivity of our analyses of the emotion recognition data, we used a multilevel longitudinal (mixed effects) model, which does not require that all participants have a measurement at all timepoints. Participants that are measured only at a single time point – such as children tested only at T1 – do not provide information about the longitudinal effects, but their data is still useful for estimating the between-subject variability and the group-level parameters at that time point. In other words, by using a multilevel modeling approach we can use all available data in a statistically principled way, rather than limiting the analysis to complete cases (i.e., to participants with a measurement at all timepoints, through listwise deletion).

**TABLE 1:** Demographic and group information for all children and separate groups across all timepoints.

**Characteristic**	**Syrian refugee experimental**	**Syrian refugee control**	**Jordanian non-refugee experimental**	**Jordanian non-refugee control**	**Total**
**T1**					
*n*	27	22	25	20	94
**Gender**					
Male	8 (30%)	11 (50%)	12 (48%)	9 (45%)	40 (43%)
Female	19 (70%)	11 (50%)	13 (52%)	11 (55%)	54 (57%)
Age [*M* (*SD*)]	8.67 (1.36)	9.96 (1.39)	8.42 (0.80)	8.6 (0.88)	8.89 (1.28)
**T2**					
*n*	16	13	22	20	71
**Gender**					
Male	3 (19%)	5 (39%)	12 (54%)	9 (45%)	29 (41%)
Female	13 (81%)	8 (61%)	10 (46%)	11 (55%)	42 (59%)
Age [*M* (*SD*)]	8.5 (1.15)	10.08 (1.19)	8.59 (0.50)	8.6 (0.88)	8.85 (1.20)
**T3**					
*n*	13	9	16	9	47
**Gender**					
Male	3 (23%)	2 (22%)	9 (56%)	4 (44%)	18 (38%)
Female	10 (77%)	7 (78%)	7 (44%)	5 (56%)	29 (62%)
Age [*M* (*SD*)]	8.54 (1.13)	10.22 (1.20)	8.63 (0.50)	8.89 (0.93)	8.96 (1.08)

**TABLE 2:** Testing and intervention details across timepoints for each subgroup of children.

**Number of children tested at each location**	**Syrian refugee experimental**	**Syrian refugee control**	**Jordanian non-refugee experimental**	**Jordanian non-refugee control**
**T1**				
School	0	0	25	20
Center	0	22	0	0
Home	27	0	0	0
**T2**				
School	0	0	22	20
Center	0	13	0	0
Home	16	0	0	0
**T3**				
School	0	0	0	0
Center	0	9	0	0
Home	13	0	16	9
Intervention location	Home	n/a	School	n/a
Testing location	Home	Awael Al Khair Community Center	Al Bonya school	Shaqa’eq Al Nouman school
Neighborhood	Sweileh	Al Hashmi Al Shamali	Sahab	Al Hashmi Al Shamali
Neighborhood description	Northern Amman, large number of residents are Syrian refugees (especially in the poorer areas), low and middle SES	Eastern Amman, large number of residents are Syrian refugees, low SES	Southern Amman (suburban area), large number of residents are Syrian refugees due to inexpensive rent, middle SES	Eastern Amman, large number of residents are Syrian refugees, low SES

*Details of testing and intervention locations and neighborhood descriptions for all subgroups. Center, Community Center (Awael Al Khair); Homes, participants’ homes; SES, socioeconomic status.*

### Reading Program

The recruitment of women (“ambassadors”) to read to the children was done through the Al Amal Community Center who advertised the WLR program. This resulted in 26 Syrian refugee and Jordanian women volunteering to become ambassadors. To become WLR ambassadors, the women were trained for 2 days by staff from the NGO Taghyeer. During the training session, the WLR ambassadors were introduced to the WLR program, had the importance of reading aloud to children explained to them, and were given instructions on how to deliver the story-telling sessions (changing vocalizations, facial expressions, and body language during reading). They were also instructed on how to gather local children for the reading program with support from the local community. After the training session, all 26 ambassadors were invited to participate in the research project, and 5 of them expressed interest in leading the experimental groups. Three of these women delivered reading sessions in their homes for Syrian refugee children in Sweileh neighborhood, whilst the remaining two were teachers delivering the sessions to the non-refugee children in the Al Bonya school in Sahab (Table 2). In the context of the emotion socialization theory, the ambassadors should be considered as teachers rather than parents, specifically influencing socialization by emotional expression modeling. Furthermore, other children in the reading groups are the peers who also influence socialization through emotion modeling by displaying natural expressive reactions to the stories.

Each ambassador was given a set of the same 20 books which were developed by WLR in consultation with writers and illustrators, as well as education, language, and psychology experts (We Love Reading, 2020a). All books were illustrated with short texts written in Arabic and were evaluated by children prior to publishing. The books were designed to be read out loud to children aged between 4 and 10 years old and cover topics relevant to the children’s lives, including stories on gender, non-violence, refugees, social inclusion, and disabilities. Each reading session lasted approximately 15 min, during which an ambassador would read one or two stories from the books provided by WLR. In order to promote uptake and engagement, WLR does not deliver mediated reading (e.g., there is no discussion of material during sessions to avoid creating a school-like environment). Although most reading-based interventions employ mediated reading, there is evidence to suggest that non-mediated reading can have positive effects on children’s development by increasing their emotional competence, knowledge, and emotion socialization abilities (Riquelme and Montero, 2013; Riquelme-Mella and García-Celay, 2016; Mahasneh et al., 2017). As planned, the WLR program was made shorter to accommodate school constraints and Ramadan, with all ambassadors providing a minimum of five reading sessions to the children. Reading groups varied in size between 5 and 20 children, with larger groups in the schools. Attendance was not recorded for all sessions. Children forming the control groups (Syrian and Jordanian) came from different neighborhoods and were recruited directly either through their school (Jordanian group) or the local community center (Syrian group).

### Experimental Tasks

#### (1) Emotion Recognition

**FIGURE 2:**
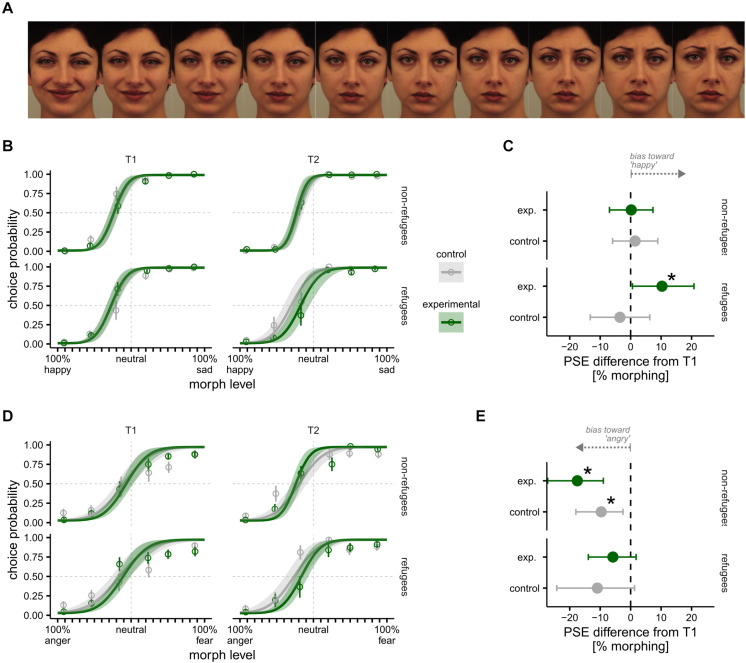
Emotion recognition bias task results between T1 and T2. **(A)** Example of a subset facial stimuli morphed between happy and sad in steps of 10% from Karolinska Directed Emotional Faces (KDEF); KDEF image ID: AF23HAS, AF23NES, AF23SAS. **(B)** Happy–sad psychometric functions presented separately for refugee (bottom) and non-refugee (top) children in control and experimental groups at T1 (left) and T2 (right). **(C)** T2 Difference in happy–sad point of subjective equality (PSE) relative to T1 in refugee (bottom) and non-refugee (top) children in control and experimental groups. **(D)** Anger–fear bias presented separately for refugee (bottom) and non-refugee (top) children in control and experimental groups at T1 (left) and T2 (right). **(E)** Difference in anger–fear PSE from T1 to T2 for refugee (bottom) and non-refugee (top) children in control and experimental groups. Asterisk indicates significant difference from T1 to T2. All error bars and bands indicate 95% Bayesian CI.

At the time of testing, there were no Middle Eastern faces databases available therefore we used Caucasian facial stimuli taken from the Karolinska Directed Emotional Faces (Lundqvist et al., 1998) and Moroccan facial stimuli from the Radbound Faces Database (Langner et al., 2010). We note however that we were interested in differences in emotion recognition across groups and timepoints, therefore any “other race” effects that might arise from not using Middle Eastern faces should affect all children equally. Faces were cropped to remove most hair and accessories (e.g., necklaces, earrings) and we created facial morphs between two extreme expressions [happy–sad (HS) and fear–anger (FA)] in steps of 10%. This resulted in 21 facial morphs (Figure 2A) on each emotion dimension (HS and FA). Morphs were created using four different face identities from the above databases (two male and two female) and were used throughout the testing, except at T3 where we included two additional facial identities (one male and one female).

The emotion recognition task was run using Matlab (Mathworks) and Psychtoolbox-3 (Brainard, 1997) and stimuli were presented on a Dell laptop computer. On a given trial, a child was shown a single facial stimulus either along the happy–sad (HS) continuum, or the fear–anger (FA) continuum and reported which expression the face displayed. Each trial began with a central fixation point that was presented for 250 ms, followed by presentation of the stimulus and then a prompt to select an emotion for that stimulus. After the child’s response a noise mask screen appeared for 250 ms and the next trial started. Children were asked to say their selection out loud and an Arabic speaking local fieldworker (blind to the task outcomes) entered their answer by pressing the relevant key. This was done to minimize key press errors, given the higher error rate for key presses in young children. Although the majority of children gave their answers in Arabic, some children wanted to demonstrate their ability to speak English and gave their answers in both Arabic and English. Stimulus presentation was controlled by a Bayesian adaptive staircase (Watson and Pelli, 1983) that stopped after 30 trials. This method selected stimuli that allowed for maximally efficient estimation of the emotion recognition bias. The bias is defined as the distance of the subjective neutral face (thus expected to yield responses equally distributed among the two emotion labels) from the mid-point of the morph continuum (the actor’s neutral face impersonation). Each child completed the emotion recognition task in two separate blocks, once for HS and once for FA and was randomly tested with a female face on one dimension and a male face on the other. The order of HS or FA was counterbalanced across children. Baseline data (T1) was collected shortly after training the WLR ambassadors and prior to beginning the reading sessions. T2 data was collected after the end of the reading program, and T3 data was a 2-month follow-up after the end of the WLR program.

#### (2) Trauma and Psychopathology Measures

Children were given locally validated questionnaires that were either originally developed in Arabic or validated in Arabic and administered orally by an Arabic-speaking fieldworker who input the responses directly into the Qualtrics Offline Application (Qualtrics, Provo, UT, United States). Following collection of demographic information, we used the questionnaires to assess children’s trauma exposure, mental health, and wellbeing.

Trauma scores were measured using the Traumatic Events Checklist (TEC) (Panter-Brick et al., 2009), which consists of 21 yes/no items covering events pertinent to situation in Syria and differentiating between direct personal experience from being a witness to an event, such as having lived in a refugee camp, having witnessed or experienced torture, and having directly witnessed a bombardment or rocket explosions related to war. Due to the challenging nature of the questions and the young age of some of the children, and in line with previous studies (e.g., Bean et al., 2006; Hall et al., 2014), the questionnaire was administered to parents or guardians of participants. Scores were summed up giving a total trauma events experienced score for each participant. Those participants with trauma scores of 1 and above (i.e., those children who had experienced at least one traumatic event) were assessed for PTSD symptoms using the Child Revised Impact of Events Scale (CRIES 8) (Perrin et al., 2005). Participants who scored 0 on the Trauma Events Checklist (i.e., those children who had not experienced any traumatic events) were given a score of 0 on the CRIES. CRIES measures symptoms of PTSD where items are scored on a scale ranging from 0 (not at all) to 5 (often), with higher scores indicating greater symptom severity (eight items, Cronbach’s α = 0.92). Items in this measure include questions relating to a traumatic event, such as: ‘Do you think about it even if you don’t mean to?,’ ‘Do you try to remove it from your memory?’ Total possible scores range from 0 to 40, with scores of 17 and above indicating symptoms consistent with the clinical criteria for PTSD (Perrin et al., 2005).

Depression and anxiety were assessed using the Arab Youth Mental Health Scale (AYMHS) (Mahfoud et al., 2011). The AYMH consists of 21 items assessed on a three-point Likert scale, ranging from rarely (1) to always (3), and results in a total score for anxiety/depression (21 items, Cronbach’s α = 0.86). A higher score indicates more symptoms of anxiety/depression. Statements in this measure pertain to children’s emotional wellbeing in the past week, such as: ‘During the last week I was upset,’ ‘During the last week I felt lonely.’ Due to the low sensitivity and specificity of the scale as a clinical screening tool, we used the symptom score rather than the cut-off, in line with recommendations from the authors of the scale’s validation study (Mahfoud et al., 2011).

The Human Insecurity and Distress Scale (HIDS) (Ziadni et al., 2011) was administered to measure scores of insecurity (10 items, Cronbach’s α = 0.73) and distress (12 items, Cronbach’s α = 0.77). HIDS uses a four-point Likert scale ranging from 1 (never) to 4 (always) with an additional response possibility (don’t know), with higher scores indicative of more insecurity or distress. HIDS includes questions such as: ‘To what extent do you worry/fear for your and your family’s future?’ for the insecurity measure, and ‘To what extent do you feel frustrated?,’ ‘To what extent do you feel angry?’ for the distress measure.

Lastly, we measured optimism using components of the Youth Life Orientation Test (YLOT) (Ey et al., 2005), which consists of four statements ranging on a scale from 0 (not true for me) to 3 (true for me), with higher scores indicating greater optimism. Examples of items in this measure are ‘I usually expect to have a good day’ and ‘When things are bad, I expect them to get better.’ The optimism scale had low reliability (Cronbach’s α = 0.50), possibly due to the small number of items.

### Analysis

To estimate bias (the morphed face level that the child perceives as neutral) for the emotion recognition tasks (HS and AF separately) we used a hierarchical Bayesian generalized linear model with a probit link function that includes a lapse rate to account for finger press errors. The advantage of hierarchical models is that by partially pooling information across participants in the same group, they can provide more robust estimates of individual psychometric parameters. This feature is particularly useful in developmental studies, in which typically children only contribute a relatively small number of trials. The model was fit in Stan (Carpenter et al., 2017) via its R interface and also included a lapse-rate parameter, corresponding to the probability of stimulus-independent responses (e.g., attention lapses or key press errors). A small subset of children (*N* = 11) in the refugee control group were mistakenly tested at T3 with a different facial identity stimulus (i.e., a different actor) than the one that was used at T1 and T2 on the emotion recognition task. To account for this potential confound, we estimated the changes in perceptual bias and sensitivity ascribable to the different actors using a separate dataset of 71 children (all Syrian refugees), which comprised all children from the Syrian refugee group at T1 (thus tested with the correct actor) plus an additional group of 22 children that were tested with the different actor. These additional 22 children were Syrian refugee children tested at T3 and recruited from the same school as the main cohort. Note that the additional group of children did not take part in the reading program, performed the task only once and with the different actor, and was well matched both in terms of age, *F*(1,69) = 0.001, *p* = 0.925, as well as gender, χ^2^(1) = 0.96, *p* = 0.33. This enabled us to estimate the effect of the different actor on bias and sensitivity whilst controlling for possible confounds due to repeating the task more than once. The analysis did not reveal reliable differences on either the bias (mean difference expressed in% morphing: 9.32, 95% CI [−2.09, 20.11]) or the sensitivity (mean difference expressed in% morphing: −0.34, 95% CI [−4.16, 3.25]). The posterior distribution over these differences was used (using a normal approximation) to derive informative priors about the effect of the different actor that was used in the analysis of the main dataset (which otherwise would not provide enough information to constrain the effect of the different actor). This approach allows adjusting our analysis for the possible effect of the different actor identity while also taking into account the uncertainty about the true size of such effect: the uncertainty is propagated from the prior to the posterior and reflected in the credible interval around our estimates at T3.

As responses to the questionnaire measures obtained at T1 were not normally distributed with the scores on an ordinal scale (which does not allow for unambiguous numerical interpretation), they were compared across the groups (Syrian refugee vs. Jordanian non-refugee at baseline to address the first research question), and across the subgroups (Syrian refugee experimental vs. Syrian refugee control, and Jordanian non-refugee experimental vs. Jordanian non-refugee control) using a Mann–Whitney *U* test. To address the second research question, longitudinal tests for changes in the questionnaires across the timepoints by intervention group were performed using a non-parametric variance analysis for longitudinal data (Brunner and Puri, 2001), using the R package nparLD (Noguchi et al., 2012). We used Spearman’s correlations to determine whether higher symptoms of PTSD, anxiety, depression, insecurity and distress, and lower optimism scores are correlated with trauma exposure, as well as to determine correlations between questionnaire scores and emotion recognition bias (to address the third research question).

### Ethics Statement

The project was granted ethical approval from the Queen Mary Research ethics board (QMERC 2018/54). All data was confidential and pseudo-anonymized. Parents/guardians gave their informed consent prior to the children’s inclusion in the study, and all children provided verbal assent.

## Results

Attrition between T2 and T3 was high due to summer holidays, with only 50% of T1 participants taking part in the last data collection. Therefore, to evaluate the WLR program, our main analysis focuses on the longitudinal comparisons between T1 and T2 within the Syrian refugee and Jordanian non-refugee groups separately. Nevertheless, for completeness, we also present comparisons between the T1 baseline and the T3 follow-up at the end of each section.

### Emotion Recognition Bias Task

#### Baseline (T1)

In these analyses, we test the first research question, evaluating differences in emotion recognition between the Syrian refugees and Jordanian non-refugees.

##### Happy–sad task

The bias or the *point of subjective equality* (PSE): We find no significant differences between the Syrian refugee and Jordanian non-refugee groups, nor between the subgroups of Syrian refugee experimental and Syrian refugee control, or between Jordanian non-refugee experimental and Jordanian non-refugee control at T1 (Table 3), with all participants displaying a significant bias toward perceiving neutral faces as sad (Figure 2B).

**TABLE 3:** Results from emotion recognition bias tasks and questionnaires across timepoints for the Syrian refugee and Jordanian non-refugee subgroups separately.

	**Syrian**	**Syrian**	**Jordanian**	**Jordanian**
	**refugee**	**refugee**	**non-refugee**	**non-refugee**
**Variable**	**experimental**	**control**	**experimental**	**control**
**T1**				
*n*	27	22	25	20
TEC	5.67(3.61)**	9.86(2.7)**	0.20 (0.5)	0.15 (0.37)
CRIES	8.96 (11.55)	12.36 (12.84)	1.04 (5.1)	2.90 (9.07)
AYMHS	28.33 (7.27)	28.57 (5.87)	27.96 (4.67)	28.35 (7.17)
HIS	21.12 (5.17)	23.10 (5.2)	23.96 (4.82)	23.95(6.51)
HDS	18.31 (5.27)	18.14 (4.62)	19 (6.31)	17.58 (5.31)
YLOT	10.26 (1.85)	10.14 (2.36)	10.52(1.58)*	11.50(1.0)*
HS bias	−27.97(11.36)	−25.09(15.35)	−22.50(9.04)	−24.36(8.31)
FA bias	5.07 (51.77)	−10.76(29.79)	−1.20(30.32)	−8.95(19.51)
**T2**				
*n*	16	13	22	20
AYMHS	31.27 (7.55)	27.75 (5.94)	27.84 (5.79)	31.13 (8.56)
HIS	23.73 (5.69)	25.33 (8.18)	23.52 (6.02)	25.58 (7.16)
HDS	23.4 (9.12)	19.27 (6.05)	19.15 (6.71)	20.45 (7.51)
YLOT	8.27 (3.26)	9.77 (2.56)	10.29 (1.68)	10.68 (1.34)
HS bias	−18.15(13.68)	−28.80(19.41)	−22.60(6.75)	−22.64(6.65)
FA bias	−11.57(21.59)	−28.62(20.73)	−24.23(9.97)	−21.72(17.40)
**T3**				
*n*	13	9	16	9
AYMHS	30 (7.83)	26.44 (5.32)	26(3.82)*	30(2.94)*
HIS	22.89 (5.65)	24.83 (5.23)	21.64(4.88)*	26.83(2.14)*
HDS	23.92 (8.16)	18 (6.52)	16.88(4.95)**	23.14(5.31)**
YLOT	9.33 (2.06)	9.56 (1.74)	10.44 (1.59)	11 (1.16)
HS bias	−26.28(13.58)	−22.50(17.23)	−21.85(8.54)	−21.02(7.14)
FA bias	−4.17(37.75)	−20.46(23.57)	−11.52(13.35)	−17.76(11.45)

*Means and standard deviations for all measures across different timepoints, presented separately for each subgroup of children. TEC, Traumatic Events Checklist; CRIES, Child Revised Impact of Events Scale (PTSD measure); AYMHS, Arab Youth Mental Health Scale (anxiety/depression measure); HIS, Human Insecurity Scale; HDS, Human Distress Scale; YLOT, Youth Life Orientation Test (optimism measure); HS, happy–sad emotion recognition bias; FA, fear–anger emotion recognition bias. ^∗^ indicates significant difference between experimental and control subgroups *p* < 0.05. ^∗∗^ indicates significant difference between experimental and control subgroups *p* < 0.01.*

##### Fear–anger task

There were no significant differences between the Syrian refugee and Jordanian non-refugee groups, nor between the experimental and control subgroups within Syrian refugees or within Jordanian non-refugees at T1 (Table 3), with children exhibiting no significant bias toward either emotion (Figure 2D).

#### Change From T1 to T2

In these analyses, we test the second research question, evaluating whether the intervention had an impact on emotion recognition for the Syrian refugees or for the Jordanian non-refugees separately from T1 to T2. Specifically, we look within groups over time, testing whether there is a difference in change in emotion recognition between the Syrian refugee controls v. Syrian refugee experimental group, and between the Jordanian non-refugee controls v. Jordanian non-refugee experimental group.

##### Happy–sad task

At T2, the Syrian refugee experimental group were significantly less biased toward sadness compared to their bias measured at T1 (Figure 2C), suggesting a possible positive effect of the WLR intervention amongst this group. There were no differences between bias measured at T1 compared to T2 within any other subgroups.

##### Fear–anger task

There was a significant increase in bias toward fearful facial expressions between T1 and T2 in both the Jordanian non-refugee experimental and control children, with no change in the Syrian refugee experimental or control children (Figure 2E).

#### Follow Up (From T1 to T3)

##### Happy–sad task

The effect of WLR on the happy–sad bias in the Syrian refugee experimental group disappeared when tested at T3 (i.e., the perceptual bias measured at T3 did not differ significantly from that measured at T1). However, the large attrition rate in each group at the last timepoint (*n* = 47 in total, with as few as nine children in some groups) makes it difficult to draw any conclusions about the persistence or not of the effect at this timepoint.

##### Fear–anger task

There were no significant differences in the fear–anger task tested at T3 compared to T1 in any of the subgroups. The shift of the bias toward fear in both Jordanian non-refugee groups at T2 disappeared at T3.

### Questionnaires

#### Baseline (T1)

In these analyses, we test the first research question, evaluating differences in trauma exposure, mental health, and wellbeing between the Syrian refugees and Jordanian non-refugees.

##### Traumatic Events Checklist (TEC) and PTSD

Traumatic Events Checklist (obtained from the parents) and CRIES responses were collected at baseline (T1) only. As would be expected, Syrian refugee children had experienced many more traumatic events (*n* = 49, *M* = 7.55, *SD* = 3.64) than Jordanian non-refugee children (*n* = 45, *M* = 0.18, *SD* = 0.44, *U* = 82, *p* < 0.001). Spearman’s ranked correlation analysis showed that among the Syrian refugee group, trauma scores were significantly correlated with age, where more traumatic events were reported for older children, *r*_*s*_(49) = 0.343, *p* = 0.008. TEC scores were also significantly negatively correlated with amount of time spent away from Syria in the refugee children, whereby the less time they had been in Jordan, the higher their trauma score, *r*_*s*_(46) = −0.317, *p* = 0.016 (Table 4). Unexpectedly, we found that trauma scores were not correlated with PTSD, anxiety, depression, insecurity, distress, or optimism levels (Table 4). There was a significant difference between Syrian refugee experimental and Syrian refugee control groups in terms of trauma exposure, *U* = 510, *p* < 0.001, with higher trauma in the Syrian refugee control group (Table 3).

**TABLE 4:** Correlations for questionnaire scores in Syrian refugee group.

**Variable**	**1**	**2**	**3**	**4**	**5**	**6**	**7**	**8**	**9**
1. TEC	–								
2. CRIES	0.096	–							
3. AYMHS	–0.140	0.270*	–						
4. HIS	0.096	0.207	0.417**	–					
5. HDS	0.283	0.287*	0.709**	0.301*	–				
6. YLOT	0.134	–0.216	−0.337*	−0.290*	−0.277*	–			
7. Age	0.343**	0.493**	0.261*	0.171	0.147	–0.141	–		
8. Time away from Syria	−0.234*	−0.340*	–0.022	−0.304*	0.036	0.251*	−0.705**	–	
9. Poverty	–0.081	–0.093	0.054	0.158	0.006	0.107	0.197	–0.068	–

*Results of Spearman’s correlations between trauma and mental health measures in Syrian refugee children. TEC, Traumatic Events Checklist; CRIES, Child Revised Impact of Events Scale (PTSD measure); AYMHS, Arab Youth Mental Health Scale (anxiety/depression measure); HIS, Human Insecurity Scale; HDS, Human Distress Scale; YLOT, Youth Life Orientation Test (optimism measure). ^∗^ indicates significant correlation *p* < 0.05. ^∗∗^ indicates significant correlation *p* < 0.01.*

Similarly, there was a significant difference between Syrian refugee (*n* = 48, *M* = 10.5, *SD* = 12) and Jordanian non-refugee (*n* = 44, *M* = 1.89, *SD* = 7.1) children in the continuous scores of post-traumatic stress symptoms, *U* = 615, *p* < 0.001. There was also a significant difference in the dichotomized PTSD score, *U* = 710, *p* < 0.001, with 40% of the Syrian refugee children experiencing post-traumatic stress symptoms in line with a diagnosis of PTSD (17 or above on the CRIES, Perrin et al., 2005), as compared to the Jordanian non-refugee participants, none of whom scored 17 or over on the CRIES. Spearman’s correlations showed that the continuous scores of post-traumatic stress symptoms were significantly correlated with age in the Syrian refugee group, with older children reporting higher levels of PTSD symptoms, *r*_*s*_(45) = 0.493, *p* < 0.001, and with years spent away from Syria, with less time living in Jordan correlating to higher PTSD scores, *r*_*s*_(42) = −0.340, *p* = 0.014 (Table 4). All correlation analyses of questionnaire scores are presented in Table 4 for the Syrian refugee group and in Table 5 for the Jordanian non-refugee group. There were no differences between the Syrian refugee experimental and control groups in PTSD scores, *U* = 318.5, *p* = 0.47 (Table 3).

**TABLE 5:** Correlations for questionnaire scores for Jordanian non-refugee group.

**Variable**	**1**	**2**	**3**	**4**	**5**	**6**
1. AYMHS	–					
2. HIS	0.304*	–				
3. HDS	0.479**	–0.037	–			
4. YLOT	−0.295*	−0.272*	−0.376**	–		
5. Age	0.076	–0.026	–0.028	–0.026	–	
6. Poverty	–0.124	0.056	–0.084	0.068	0.385**	–

*Results of Spearman’s correlations between trauma and mental health measures in Jordanian non-refugee children. TEC, Traumatic Events Checklist; CRIES, Child Revised Impact of Events Scale (PTSD measure); AYMHS, Arab Youth Mental Health Scale (anxiety/depression measure); HIS, Human Insecurity Scale; HDS, Human Distress Scale; YLOT, Youth Life Orientation Test (optimism measure). ^∗^ indicates significant correlation *p* < 0.05. ^∗∗^ indicates significant correlation *p* < 0.01.*

##### Anxiety and depression

Anxiety and depression scores did not differ significantly between Syrian refugees (*n* = 45, *M* = 28.4, *SD* = 6.6) and Jordanian non-refugees (*n* = 45, *M* = 28.1, *SD* = 5.8; *U* = 1000.5, *p* = 0.90). There were also no differences between Syrian refugee experimental and control subgroups, *U* = 274, *p* = 0.62, or between Jordanian non-refugee experimental and control subgroups at baseline, *U* = 234.5, *p* = 0.72 (Table 3).

##### Insecurity and distress

Neither insecurity nor distress scores differed between Syrian refugees (insecurity: *n* = 46, *M* = 22, *SD* = 5.2; distress: *n* = 48, *M* = 18.2, *SD* = 4.9) and Jordanian non-refugees (insecurity: *n* = 44, *M* = 23.4, *SD* = 5.6; *U* = 1153.5, *p* = 0.25; distress: *n* = 44, *M* = 18.4, *SD* = 5.8; *U* = 1036, *p* = 0.99). There were also no differences between Syrian refugee experimental and control participants (insecurity: *U* = 302, *p* = 0.35; distress: *U* = 291, *p* = 0.92), nor between Jordanian non-refugee experimental and control participants (insecurity: *U* = 265.5, *p* = 0.55; distress: *U* = 194, *p* = 0.3) at T1 (Table 3).

##### Optimism

There were no significant differences in optimism scores between Syrian refugees (*n* = 49, *M* = 10.2, *SD* = 2.1) and Jordanian non-refugees (*n* = 45, *M* = 10.9, *SD* = 1.4; *U* = 1314, *p* = 0.09) groups, of between the Syrian refugee experimental and control subgroups at T1, *U* = 311, *p* = 0.77. The Jordanian non-refugee experimental group had a significantly lower score of optimism than Jordanian non-refugee control group, *U* = 343, *p* = 0.02 at baseline (Table 3).

#### Change From T1 to T2

**FIGURE 3:**
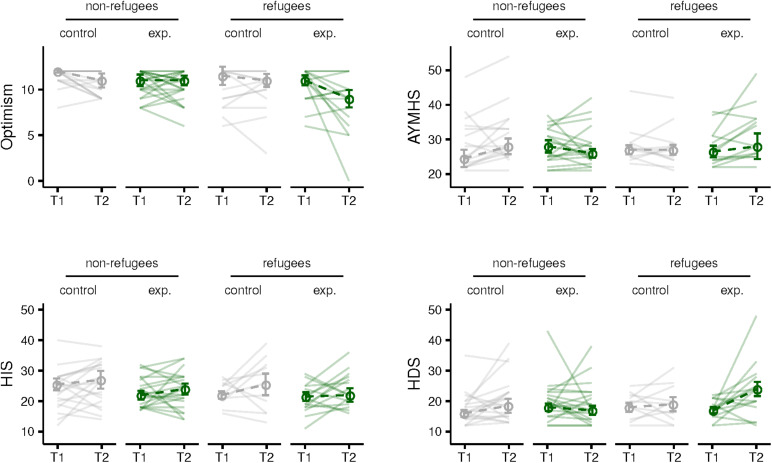
Results from psychopathology questionnaires between T1 and T2 presented separately for refugee and non-refugee children in control and experimental groups. Optimism (top left), anxiety and depression measured by Arab Youth Mental Health Scale (AYMHS) (top right), Human Insecurity Scale (HIS) (bottom left), and Human Distress Scale (HDS) (bottom right).

There were no statistically significant changes in any of the mental health measures between T1 and T2 for any of the subgroups (Table 6 and Figure 3).

**TABLE 6:** Change in questionnaire scores from T1 to T2 and at follow-up (from T1 to T3).

**Variable**	**Syrian refugees**			**Jordanian non-refugees**		
	***F***	***df***	***p***	***F***	***df***	***p***
**T1–T2**						
AYMHS	5.66^a^	1	0.02	2.06	1	0.15
HIS	0.16	1	0.69	0.51	1	0.47
HDS	3.70	1	0.05	3.32	1	0.07
YLOT	3.06	1	0.08	1.59	1	0.21
**Follow up (T3)**						
AYMHS	2.12	1.47	0.13	2.23	1.85	0.11
HIS	0.11	1.38	0.82	3.28^b^	1.99	0.04
HDS	2.05	1.42	0.14	5.88^c^	1.84	0.00
YLOT	2.40	1.89	0.09	0.16	1.79	0.83

*Non-parametric variance analysis results (Noguchi et al., 2012) for changes between timepoints in questionnaire measures for Syrian refugee subgroups (experimental vs. control) and Jordanian non-refugee subgroups (experimental vs. control) separately. Statistically significant changes in timepoints are then investigated within each subgroup using Wilcoxon *post hoc* analysis (comparing each timepoint to the T1 baseline). Note that all effects became insignificant after correcting for multiple comparisons. ^*a*^Wilcoxon signed rank *post hoc* tests revealed a significant increase in AYMHS only in the Syrian refugee experimental subgroup from T1 to T2, *W* = 6.5, *p* = 0.02. However, after controlling for multiple testing with Bonferroni correction, this comparison became non-significant, *p* = 0.08. ^*b*^Wilcoxon signed-rank tests failed to show any significant differences in insecurity scores from baseline to T3 (non-corrected *p* > 0.14). ^*c*^Wilcoxon signed-rank tests failed to show any significant differences in distress scores from baseline to T3 (non-corrected *p* > 0.08). AYMHS, Arab Youth Mental Health Scale (anxiety/depression measure); HIS, Human Insecurity Scale; HDS, Human Distress Scale; YLOT, Youth Life Orientation Test (optimism measure). *F*, ANOVA-type statistic; *df*, degrees of freedom.*

#### Follow Up (From T1 to T3)

We found no significant changes in any of the questionnaire scores for any of the subgroups at T3 follow up (Table 6). Note that the high attrition rate at the third timepoint (see Table 1) is particularly problematic for the questionnaire results.

### Emotion Recognition Bias and Questionnaires

To assess our third research question of whether survey measures of mental health and wellbeing were related to performance on emotion recognition tasks, we have conducted a series of Spearman’s correlations.

#### Happy–Sad Task

Spearman’s correlations showed significant correlation between HS bias and scores on continuous PTSD scale [*r*_*s*_(89) = −0.21, *p* = 0.04], distress [*r*_*s*_(89) = 0.24, *p* = 0.02] and optimism [*r*_*s*_(91) = 0.29, *p* = 0.006] for all participants combined. However, after controlling for multiple testing using Bonferroni correction, correlations of HS bias with PTSD and distress became non-significant (*p* = 0.24 and *p* = 0.12, respectively). Correlation between bias and optimism scores remained statistically significant after multiple testing correction, *p* = 0.034, indicating that children who scored higher on optimism had a smaller bias toward sad facial expressions (i.e., they were less likely to indicate that neutral faces were sad).

#### Fear–Anger Task

Spearman’s ranked correlations did not show any statistically significant associations between the FA bias and questionnaire scores for all participants.

## Discussion

Our aim in this project was threefold: *first*, using a method that bypasses self-inspection or parent report, we measured whether emotion recognition was affected in Syrian refugee children compared to Jordanian non-refugee children. *Second*, we examined whether taking part in a reading-based intervention would reduce emotion recognition biases toward negative facial expressions and improve mental health in refugee and non-refugee children living in Jordan. *Third*, we examined how any atypicalities in emotion recognition relate to self-reported mental health.

Contrary to the first hypothesis, we found that both Syrian refugee and Jordanian non-refugee children were equally biased toward perceiving neutral expressions as sad at baseline. With respect to self-reported mental health, Syrian refugee children were exposed to significantly more traumatic events and had higher levels of PTSD than Jordanian non-refugees, but no other mental health outcomes differed between the groups. Furthermore, the questionnaires scores were not significantly correlated with the bias for sad facial expressions, with the exception of a weak correlation between optimism and the bias. Our results differ from earlier findings, where facial emotion perception has been linked to emotional wellbeing and mental health (Stuhrmann et al., 2011; Zwick and Wolkenstein, 2017), and has been shown to be impaired in children with a history of early life adversity (Pollak et al., 2000; Pollak and Tolley-Schell, 2003; Herringa, 2017). However, it is worth noting that war trauma and displacement as a form of early adversity have not yet been investigated in relation to emotion processing. Indeed, most studies on impaired emotion recognition focus on childhood maltreatment, but it is likely that these interpersonal types of early adversity have a different impact on emotion recognition than experiences of war trauma and displacement. Our results also suggest that there are other factors which might influence the mechanisms of emotion recognition. Surprisingly, we found no bias in the fear–anger task in either Syrian refugees or Jordanian non-refugees, however this may reflect the fact that both of these emotions are negatively valanced. Since previous studies showed both attention (Pollak and Sinha, 2002; Romens and Pollak, 2012) and avoidance (Pine et al., 2005) toward threat in children with a history of early adversity, a more nuanced approach might be needed to investigate threat processing mechanisms in war-affected children.

We found a reduction in the sad bias on the emotional expression task immediately after the end of the intervention only in the Syrian refugee children who took part in the WLR program. These findings are partially consistent with previous work which shows benefits of shared book reading on emotional development and wellbeing in children and adolescents (Adrian et al., 2005; Daunic et al., 2013; Kumschick et al., 2014), although the lack of improvement in Jordanian non-refugee children is surprising. It is possible that children who experienced early life adversity could be more receptive to this type of non-mediated reading intervention than their peers without experiences of trauma and displacement, in line with Finlon et al. (2015). The positive effects of the reading program on the sad bias for Syrian refugees disappeared at the 2-month follow-up, suggesting a relatively short-term effect, although the high attrition rates at the third testing timepoint preclude any conclusive judgments about potential longer-term effects. Nonetheless, these results suggest that reading-based programs can influence how children interpret and recognize adult facial expressions. With increasing numbers of refugee and other forcibly displaced children worldwide (UNHCR, 2020), accessible and cost-effective programs which do not require substantive resources or trained clinicians are needed to address children’s healthy emotional and cognitive development. These preliminary emotion recognition results suggest a possible positive role of the WLR intervention in shaping emotional competence in Syrian refugee children, although longer term impacts would need to be rigorously evaluated.

One possible explanation for the finding that the Jordanian non-refugee children did not show improvements in their sad bias following the WLR program may be that the location of delivery of the reading sessions affects the outcome. Syrian refugee children took part in the program delivered by local women in community centers and homes, with smaller groups tending to include their close friends and neighbors. This was the primary setting for which WLR program was developed (We Love Reading, 2020b): it does not rely on aligning with the school academic year and can be delivered in humanitarian contexts where access to schools is limited or non-existent. Reading sessions for the Jordanian non-refugee children were delivered by teachers in school during their class time to larger groups of children. Based on anecdotal reports from the teachers and children to the fieldworkers, it is possible that the classroom environment might negatively impact the effectiveness of the program, by creating a more rigid setting in which children felt the participation was obligatory as part of the curriculum and that their performance was being evaluated. Stressful school environments increase children’s self-reported anxiety (Piekarska, 2000), thus the potential benefits of the program might have been diminished in the Jordanian non-refugee experimental group as a result of the difference in setting. The fact that the Jordanian non-refugee children did not show improvements following the WLR program tentatively suggests that the location of delivery of the reading sessions, as well as the larger group sizes, may affect the outcome (although see Dajani et al., 2019). Indeed, the majority of the successful reading interventions mentioned previously took place outside of a school setting. Future studies could explore whether the location of WLR delivery and size of WLR group plays a significant role in the final outcome when participants in all groups are comparable in all other aspects.

We found a significant difference in emotion recognition bias in the fear–anger task amongst all Jordanian non-refugee children in both experimental and control group at the second timepoint in April, with these children showing a significantly larger bias toward perceiving expressions as fearful rather than angry. This result disappeared at T3 (in June, 2 months after the end of the program) suggesting a temporary effect, although low participant numbers at this timepoint make it difficult to draw concrete conclusions. As mentioned above, we believe that these results may have been affected by the group difference in testing environments. As T2 took place just before the exam period, the school environment might have induced exam anxiety resulting in an increased fear bias in the Jordanian non-refugee children compared to Syrian refugee children tested outside of the school environment (although we note that the questionnaires did not show increased anxiety scores in Jordanian non-refugee children). Interestingly, acute socio-evaluative stress has been shown to have similar effects of heightened sensitivity to fearful faces in primary school children (Chen et al., 2014). This interpretation is consistent with the return of the fear bias to baseline levels in Jordanian non-refugee children at T3, as T3 data collection occurred during summer holidays and all children were tested in their homes.

A key limitation of the study was the non-randomized control trial approach. One possible outcome of not having complete participant randomization is that the Syrian refugee experimental and Syrian refugee control groups differed in their exposure to trauma, with the control group exhibiting higher trauma scores, although child reports of the trauma outcome as measured on PTSD symptoms showed no group differences. It is possible that this difference in trauma scores reflect war and displacement events that only the mothers recalled, and of which the children were not consciously aware. For instance, primary school children were previously shown to under-report war events compared to caregivers’ reports (Goldin et al., 2003). Although it is also possible that children may have experienced more trauma than their parents are aware of, and therefore the trauma scores could be under-reported. Another limitation was the high attrition rate, especially at T3 (2 months after the intervention), with the highest drop-out rate in the Jordanian non-refugee control group. This was mainly due to the timing of the third wave of data collection, which fell at the start of summer holidays. This lack of data prevents reliable assessment of the long-term effects of WLR on emotion recognition bias or psychopathology scores. However, using the multilevel model approach we were able to account for attrition for same time-point comparisons between groups in the emotion recognition bias task, although this was not possible for the analysis on the questionnaire data. Future studies are needed to investigate the long-term impacts of reading programs on refugee children’s mental health and wellbeing. Further research could employ additional measurements to assess the effects of a reading program; focusing more broadly on emotional health or functioning rather than diagnostic concepts of ‘anxiety’ and ‘depression’ might be more consistent with the likely impacts of this intervention. It would also be beneficial to more systematically control the books used during the reading sessions, so the material covered by all experimental groups is the same. Future research should also examine if the content of the books influences the intervention outcomes, especially when the reading program is administered to younger children. Finally, research could specifically investigate the perception of fear and anger more closely. For instance, these two emotions could be separately paired with a display of positive emotion, such as happiness, to force the choice between a positive and negative expression rather than between two negative expressions, in order to explore the strength of the fear bias, before and after socioemotional interventions.

In conclusion, we find that Syrian refugee and Jordanian non-refugee children show a bias in emotion recognition toward sad facial expressions that does not correlate with self-reported mental health. In our pilot study we also find that a reading-based intervention may have positive, short-term effects on emotion recognition in Syrian refugee children. Our findings also highlight that facial emotion recognition paradigms may be a useful method to assess emotional wellbeing in children. Our results tentatively add to the growing body of literature suggesting that reading-based programs may positively affect emotional development in children, although it remains to be determined which factors influence the program’s success (e.g., content of the books, location of delivery, person delivering the program).

## Data Availability Statement

The datasets presented in this study can be found in online repositories. The names of the repository/repositories and accession number(s) can be found below: https://osf.io/zmhbu/.

## Ethics Statement

The studies involving human participants were reviewed and approved by the Queen Mary University of London Research Ethics Board (ref. QMERC2018/54). Written informed consent to participate in this study was provided by the participants’ legal guardian/next of kin.

## Author Contributions

IM, KH, and RD designed the study. IM, JM, ML, and DA collected the data. ML conducted statistical analysis. JM, IM, KH, and ML wrote the manuscript with contributions from all authors.

## Conflict of Interest

RD is the Director of WLR. The remaining authors declare that the research was conducted in the absence of any commercial or financial relationships that could be construed as a potential conflict of interest.
